# Characterization and Seasonal Dynamics of Tick Populations in Dairy Cattle Production Systems of Northwestern Colombian Amazon

**DOI:** 10.3390/vetsci11060244

**Published:** 2024-05-29

**Authors:** Cesar A. Zapata, Edna G. O. Morea, Dúber A. Mora-Motta, Diana M. M. Ojeda, Esther J. Quiceno-Mayo, Diego A. Toro, Fausto A. Ortiz-Morea

**Affiliations:** 1 Centro de Investigaciones Macagual CIMAZ-MACAGUAL, Universidad de la Amazonia, Florencia 180002, Colombia; c.zapata@udla.edu.co (C.A.Z.); edna.gicela-ortiz-morea@unesp.br (E.G.O.M.); du.mora@udla.edu.co (D.A.M.-M.); dianamotta121314@gmail.com (D.M.M.O.); quicenoesther560@gmail.com (E.J.Q.-M.); diegoalextoro@gmail.com (D.A.T.); 2 Ciencias Naturales y Desarrollo Sustentable, Facultad Ciencias Agropecuarias, Universidad de la Amazonia, Florencia 180001, Colombia; 3 El Centro de Investigaciones e Innovación Uninavarra, Fundación Universitaria Navarra UNINAVARRA, Facultad de Salud, Neiva 410010, Colombia

**Keywords:** *Rhipicephalus microplus* complex, livestock, Amazonia, *Bos primigenius taurus*, *Bos primigenius indicus*

## Abstract

**Simple Summary:**

In the northwestern Colombian Amazon, ticks are a significant issue for dairy cattle in the northwestern Colombian Amazon, affecting the animals’ health and the productivity of farms. Our study aimed to identify the most common types of ticks in this area and how environmental factors such as temperature and rainfall affect their numbers. We discovered that only one kind of tick dominates in this region. Interestingly, we found that these ticks are more abundant during warmer and drier months, especially affecting a certain type of cattle more than others. Based on our findings, we recommend that farmers implement tick control measures directly on the cattle and in the pastures where these animals graze more frequently during warmer periods. This dual approach is crucial for effectively managing tick populations, promoting animal health, and ensuring dairy farms’ efficient operation. This research is valuable for local farmers and could also benefit agricultural practices in similar environments worldwide.

**Abstract:**

Cattle ticks are a significant health concern in tropical livestock production due to their hematophagous behavior and potential as vectors for human and animal pathogens. In this study, we investigated the tick population present in dairy cattle production, calves, and grazing areas of livestock systems in the northwestern Colombian Amazon. Identification was based on taxonomic keys and molecular markers. Phylogenetic relationships were established using mitochondrial *COX1* and 16S genes. Population structure analysis was performed considering age, racial type (*B. indicus* vs. *B. taurus*), and the influence of environmental factors and the geomorphological landscape on tick population dynamics. Our findings revealed the presence of a single tick species, with a unique haplotype identified for each mitochondrial gene assessed. Phylogenetic analysis classified the found species within Clade A of the *Rhipicephalus microplus* complex. Ticks were more prevalent during periods of low rainfall and high temperature, and *B. taurus* cows exhibited the highest tick abundance. Thus, these results provide insights into the population characteristics and distribution of the tick species present in dairy cattle production systems in the northwestern part of the Colombian Amazon. This information is fundamental for developing targeted strategies based on seasonal variation and host characteristics to mitigate tick infestation severity in the region.

## 1. Introduction

Cattle production plays an important role in meeting the growing demand for animal-derived food products. In Colombia, the cattle industry significantly contributes to the agricultural sector and the economy. It accounts for 21.8% of the national Gross Domestic Product (GDP) within agriculture and employs 19% of the agricultural workforce [1]. The national cattle inventory is estimated at 29,42,539 animals distributed across the country (Colombian Agricultural Institute [ICA]) [2]. Notably, cattle farming has extended into the northwestern region of the Colombian Amazon, particularly in the department of Caquetá, contributing 7.2% of the national cattle inventory distributed among 20.267 farms [2]. Livestock production in this region typically involves traditional practices such as extensive grazing and utilizes cattle that are the result of crossbreeding between animals from the *Bos primigenius taurus* and *Bos primigenius indicus* racial groups. These mixed-breed cattle, often referred to as “seven colors” due to their varied coat patterns, represent an amalgamation of different breeds [3].

Cattle production is challenged by sanitary threats such as the presence of ticks [4]. Ticks are considered one of the main parasites affecting livestock production globally, especially in tropical and subtropical regions [5,6]. The tick species predominantly associated with the infestation of cattle livestock belong to the *Rhipicephalus* (Boophilus) subgenus [6,7,8]. Their hematophagous behavior not only directly compromises animal welfare but also makes them vectors for pathogenic agents such as *Babesia* “sp.” and *Anaplasma* “sp.”, leading to significant economic losses and posing a threat to public health [9,10,11]. The presence of ticks is influenced by environmental factors such as rainfall, temperature, and relative humidity, which impact its biological cycle [12,13]. Optimal environmental conditions vary depending on the altitude, ranging from sea level to 2.600 m above sea level, with annual rainfall levels between 400 and 2,800 mm per year [14]. Elevated temperatures have been linked to higher tick abundance [15], while elevated rainfall levels often hinder egg hatching [16,17].

In Colombia, the presence of Rhipicephalus (Boophilus) microplus has been documented in cattle across the Caribbean, Andean, and Orinoquia regions [18,19,20]. It has also been observed that factors such as breed, sex, age, and live weight, along with agroecological conditions, influence tick infestation rates in Colombian cattle production systems [18,21]. Despite the importance of livestock production in the northwestern region of the Colombian Amazon, limited information exists about the tick population affecting cattle in this region. This region’s warm and humid climate provides suitable conditions for the proliferation of ticks. Therefore, this study aimed to characterize the tick population in traditional cattle dairy production systems in the northwestern part of the Colombian Amazon region. We used both morphological keys and molecular markers to identify the specimens found. We assessed the infestation levels, considering factors such as the cattle racial type (*B. taurus* and *B. indicus*), age of the host (cow and calf), environmental conditions (rainfall, temperature, and relative humidity), and the landscape (hill and mountain) where the livestock system was located.

## 2. Materials and Methods

### 2.1. Description of the Study Area

The study was carried out in the northwestern part of the Colombian Amazon region, in the department of Caquetá, municipality of Florencia. This region features a tropical rainforest climate (Af type in Koppen classification), with an average annual temperature of 26.05 °C, an average annual rainfall of 3500 mm, and relative humidity levels over 80%. The study was performed in three dairy cattle production systems farms that are located in two main landscapes (hills and mountains) of the Colombian Amazon region where cattle ranching takes place [22].

**Figure 1:**
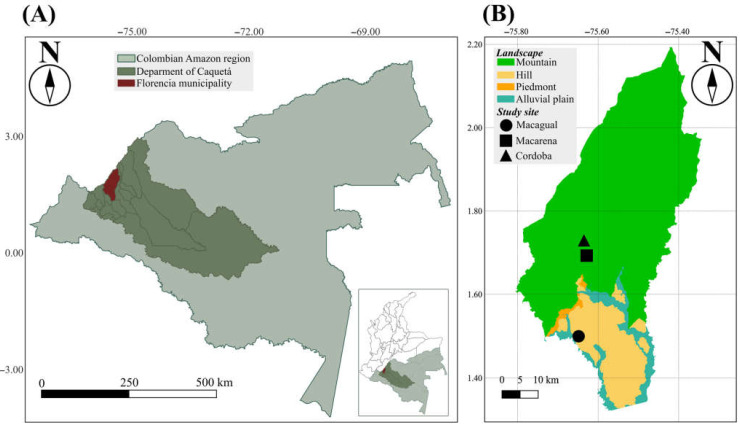
Map of the study area. (**A**) Colombia Amazon region. (**B**) Location of the study sites (Macagual, Macarena, and Cordoba) in the municipality of Florencia.

The farms include Centro de Investigaciones Macagual—CIMAZ (Macagual) (N1.501225, −75.661080), situated between 215 and 250 m above sea level (m.a.s.l.) in a hill landscape with topography that ranges from flat to slightly undulating, and Macarena (1.700, −75.61667) and Córdoba (1.713647, −75.631901) sites, located between 510 and 620 m.a.s.l. in a mountain landscape with topography that varies from steep to rolling (Figure 1). 

These farms follow typical management practices of farmers in the region, comprising nutritional and sanitary management, which include monthly acaricide treatment to control external parasites. Pastures are primarily composed of grasses such as *Urochloa* sp., *Paspalum* sp., and *Homolepis* sp., legumes such as *Calopogonium* sp., *Desmodium* sp., *Pueraria* sp. *y Stylosanthes* sp., and weeds including *Clidemia* sp., *Ceratophyllum* sp.

### 2.2. Tick Sampling Periods

In the Macagual study site, ticks were systematically sampled monthly for 12 months, from March 2020 to February 2021, thus allowing us to monitor the tick populational dynamic throughout the year. Concurrently, environmental variables such as temperature, rainfall, and relative humidity were recorded monthly in the Macagual study site to provide context for the observed tick population trends (Supplemental Table S1). 

In the Macarena and Cordoba study sites, tick sampling was strategically conducted during two climatically distinct periods: high rainfall from May to July 2020 and low rainfall from December 2020 to February 2021. This approach was designed to capture tick dynamics under constraining environmental conditions in sites located in different landscapes. 

Despite the differences in sampling periods, a uniform tick collection methodology was implemented across all three sites to ensure consistency in data collection. 

### 2.3. Tick Collection

At each study site, only animals with no pathological disorders and body condition scores (BSC) between 3 and 3.5 on a scale ranging from 1 (extremely thin) to 5 (extremely fat) were eligible for sampling. Subsequently, cattle were categorized into *B. taurus* or *B. indicus* breed groups, following the classification typology proposed by Giovambattista et al. (2000) [23]. Within each breed group, 5 lactating females aged 3 years or older (milk production phase between 4 and 7 months) and 5 calves under 1 year old were selected for tick collection. Ticks visible on the right side of each individual, with a size ≥4 mm, were collected for analysis. Tick collection was performed one day before the monthly control of external parasites to ensure the data accurately reflected natural tick abundance. The reported tick abundance per animal reflects the count of adult ticks collected solely from the right side of each animal without any extrapolation. 

Additionally, larvae were sampled in pastures where the cows in the milk production phase were grazing, employing a tick drag [24]. Briefly, a 1 m × 1 m frame covered with a white cloth canvas was dragged across the pasture surface. This process was repeated for eight sweeps, each extending 70 m. Following each sweep, larvae trapped on the canvas were counted using a magnifying glass. 

### 2.4. Tick Morphological Characterization 

Morphological characterization was conducted using established morphological keys based on the size and shape of the gnathosome, shield shape, presence or absence of eyes, festoons, ornaments, the shape of the first pair of coxae, spiracle shape, location of the anal groove and adanal plates [25,26,27].

### 2.5. DNA Extraction, PCR, and Sequencing

A total of 35 samples (16 samples from the Macagual site, 9 from Macarena, and 10 from Cordoba), each containing a pool of 5 ticks collected from the host, were used for amplification and sequencing analysis. Genomic DNA from whole tick specimens was extracted using the Molecular Biology kit (BIO BASIC USA) following the manufacturer’s instructions. 

Two mitochondrial loci were targeted for DNA amplifications: the 16S rRNA gene (470 bp) using the primer pair 16S +1 (5′-CTGCTCAATGATTTTTTAAATTGCTGTGG-3′) and 16S −1 (5′-CCGGGTCTGAACTCAGATCAAGT-3′) [28], and the COX1 gene (710 bp) with primers LCO1490 (5′-GGTCAACAAATCATAAAGATATTATTGG-3′) and HCO2198 (5′-TAAACTTCAACTTCAGGGTGACCAAAAAATCA-3′) [29].

PCR reactions were carried out in a final volume of 25 μL, containing 12.5 μL of Taq ready mix PCR, 0.7 μL (10 μM) of each primer, 1 μL of DNA (50 ng), and ultrapure water. The thermal cycling conditions included an initial denaturation at 95 °C for 5 min, followed by 35 cycles of 95 °C for 30 s, annealing at 51 °C (16 rRNA) and 50 °C (*COX1*) for 30 s, and extension at 72 °C for 1 min, with a final extension step at 72 °C for 10 min. Amplified products were visualized on 1% agarose gels stained with GelRed (Biotium, Fremont, CA, USA). Positive amplification products were purified and subjected to forward sequencing using the ABI Prism 3730 automated sequencer. 

### 2.6. Sequence Analysis

Chromatograms obtained from each amplicon were visually inspected using FinchTV 1.4.0 software (https://digitalworldbiology.com/FinchTV accessed on 27 April 2023). Sequences were manually edited to remove low-quality reads at the 5′ and 3′ ends (with a quality score below 25). The curated sequences were then individually aligned to generate consensus sequences for each locus. To confirm the identity of tick species, consensus sequences were compared with available GenBank databases using BLASTN (https://blast.ncbi.nlm.nih.gov/Blast.cgi).

### 2.7. Phylogenetic Analysis

The haplotype found in our study was compared with reference sequences sourced from GenBank. Sequences from the 16S rRNA and *COX1* genes were aligned separately using the MAFFT tool [30], and subsequent editing was performed with trimAl v.1.2 software employing the automated cut parameter (-automated1) [31]. Maximum likelihood (ML) phylogenetic analyses were conducted in MEGA11 [32], employing bootstrap testing of 1000 replicates. Evolutionary distances were calculated using the Tamura-3-parameter (T92+I) model. The resulting tree topologies were visualized using FigTree v 1.4.4 and refined using Adobe Illustrator v 2.8.0.27. *Ornithodoros rostratus* was included as an outgroup for comparative analysis.

### 2.8. Statistical Analysis 

Tick absolute abundances were analyzed using a generalized linear model (GLM) with the Poisson distribution family, which is commonly used for count data. Following model adjustment, the HSD Tukey test (*p* < 0.05) was applied to identify significative differences among mean values utilizing the “multcomp” package [33].

A redundancy analysis (RDA) was conducted to explore the correlation between climatic variables and the absolute abundances of ticks observed in the Macagual study site over a 12-month period. The statistical package “vegan” [34] was utilized for this analysis. Additionally, a Permutational Multivariate Analysis of Variance (PERMANOVA) with 999 permutations was performed to determine its significance. 

Principal Component Analysis (PCA) was utilized to explore the correlation between tick abundances and periods of low and high rainfall across all three study sites. The analysis was performed using the “FactoMineR” package [35] and the “factoextra” package [36]. Additionally, the Monte-Carlo test with 999 permutations was employed to evaluate the overall effect of rainfall seasons on tick abundances, using the “Ade4” package [37]. All analyses were carried out using R version 4.2.0 [38] and RStudio version 1.3.1 [39]. 

## 3. Results 

### 3.1. Ticks Present on Cattle in the Colombian Amazon Belong to the Rhipicephalus microplus Complex

A total of 23,690 specimens, comprising 22,409 ticks and 1281 larvae, were collected from the three study sites. In Macagual, 10,783 ticks were collected from cows, 6220 from calves, and 542 larvae from pastures. In Macarena, 1798 ticks were collected from cows, 622 from calves, and 99 larvae from pastures. Finally, in Cordoba, 1662 ticks were collected from cows, 1324 from calves, and 640 larvae from pastures. Morphologically, the ticks were identified as belonging to the R. microplus complex, exhibiting sexual dimorphism with a female-to-male ratio of 3.86 to 1, respectively.

**Figure 2:**
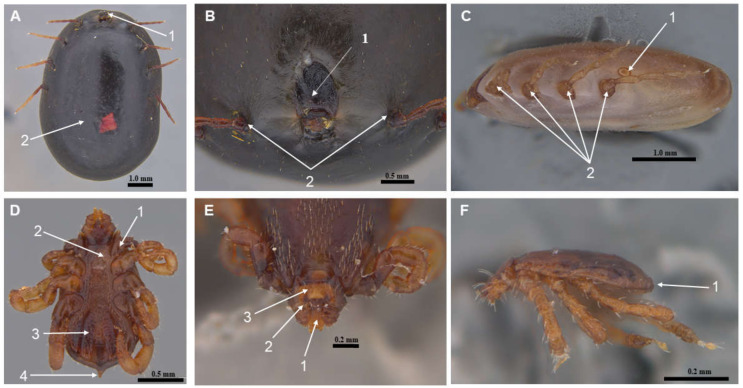
Representative photographs illustrating key morphological features of *Rhipicephalus microplus* specimens collected during the study. (**A**) Female ventral view: 1. gnathosoma; 2. idiosome. (**B**) Female frontal view: 1. shield; 2. first pair of legs. (**C**) Female lateral view: 1. spiracular plate; 2. locomotor system. (**D**) Male ventral view: 1. first coxa bifida; 2. genital opening; 3. ventral plates; 4. caudal peduncle. (**E**) Male frontal view: 1. hypostome; 2. pedipalp; 3. base of gnathosoma. (**F**) Male lateral view: 1. absence of festoons.

The characterization was conducted based on the consistent identification of the following morphological features: a short and poorly defined gnathosoma, first pair of bifid coxae, genital groove, anal opening, hypostoma, reduced palps, hexagonal capitulum base, ventral plates, shield without ornamentation, absence of scallops, and a rounded or oval spiracular plate located posterior to the last pair of coxae. In females, a marginal groove was observed only in fed individuals (Figure 2A–C), while males exhibited a caudal peduncle or terminal apophysis to the idiosoma (Figure 2D–F).

In order to accurately identify *R. microplus* ticks, molecular analyses were performed using DNA sequences of mitochondrial genes 16S rRNA and *COX1*. A total of 35 tick samples, each containing 5 individuals, were used for amplification and sequencing analysis. The coverage for the 16S rRNA gene was 400 bp, while for the *COX1* gene, it was 634 bp. Sequencing analysis revealed the presence of a unique haplotype for each gene across all examined specimens. These haplotypes have been uploaded to the GenBank database under accession code PP566877 for the 16S rRNA and PP567310 for the *COX1*. The uniformity of haplotypes across our tick samples indicates a low genetic diversity within this tick population for the assessed mitochondrial genes.

**Figure 3:**
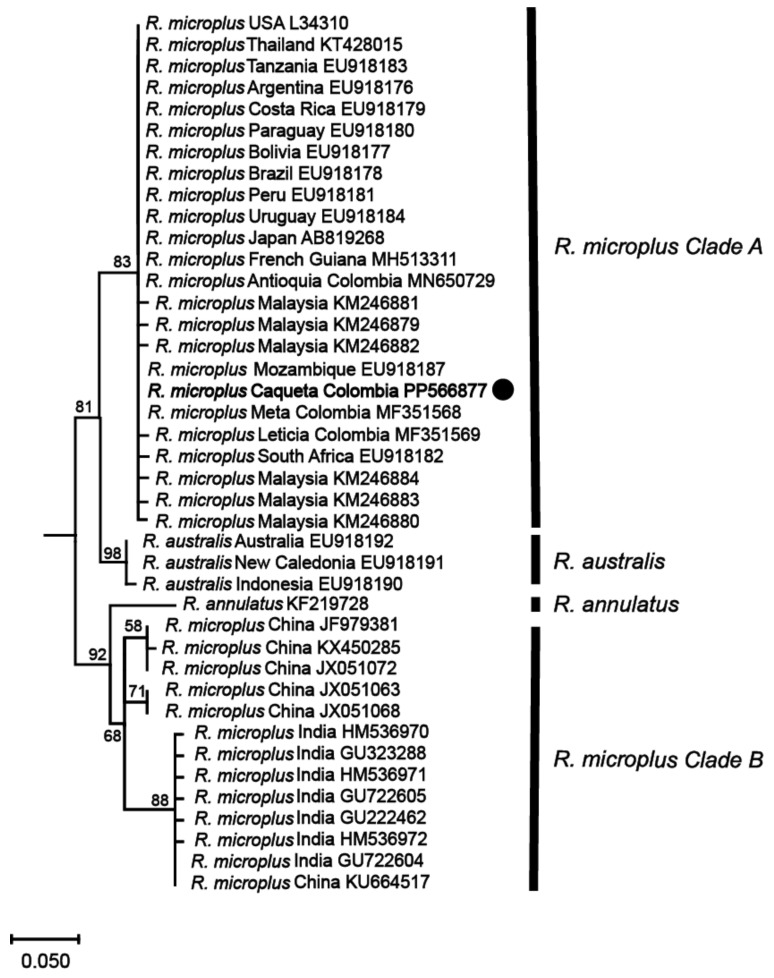
Maximum likelihood phylogenetic tree based on partial fragments of the 16S rRNA gene of *Rhipicephalus microplus*. Supporting values (1000 bootstraps) are reported at each node, and the black circle represents the study sequence. The tree is rooted with *Ornithodoros rostratus* as the outgroup, which is not shown in the figure to emphasize the *R. microplus* complex.

**Figure 4:**
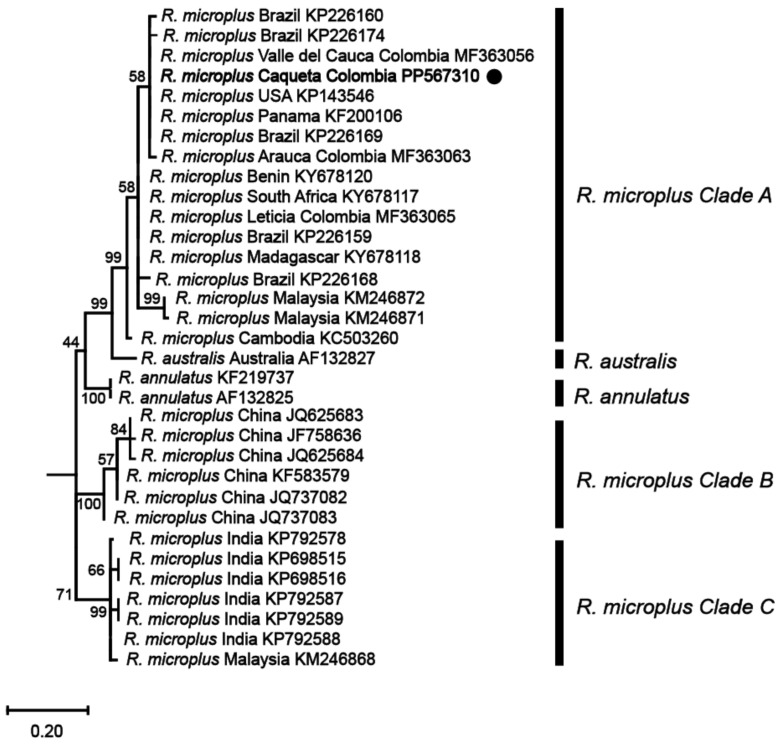
Maximum likelihood phylogenetic tree based on partial fragments of the *COX1* gene of *Rhipicephalus microplus*. Supporting values (1000 bootstraps) are reported at each node, and the black circle represents the study sequence. The tree is rooted with *Ornithodoros rostratus* as the outgroup, which is not shown in the figure to emphasize the *R. microplus* complex.

Phylogenetic analyses based on these mitochondrial markers were then conducted to determine the genetic relationships among the identified *R. microplus* haplotypes and reference isolates of *R. microplus*, *R. australis*, and *R. annulatus* from different regions around the world. The analyses revealed that the haplotype from the northwestern region of the Colombian Amazon (*R. microplus* Caquetá Colombia) was clustered within *R. microplus* Clade A, which includes a diverse range of isolates from various regions such as Malaysia, Thailand, Japan, Africa, and the Americas (Figure 3 and Figure 4, and Supplemental Figures S1 and S2). 

The phylogenetic tree constructed from the 16S rRNA gene (Figure 3) exhibited a topology consistent with previous studies on the *R. microplus* complex [7,8], revealing the genetic diversity within the species. Similarly, the phylogenetic analysis based on the *COX1* gene (Figure 4) sequences also presented a topology consistent with the *R. microplus* complex delineated in previous studies [7,8], further confirming the genetic diversity within the species.

### 3.2. Host Race, Age, and Seasonal Dynamics Shape Tick Infestation Severity

**Figure 5:**
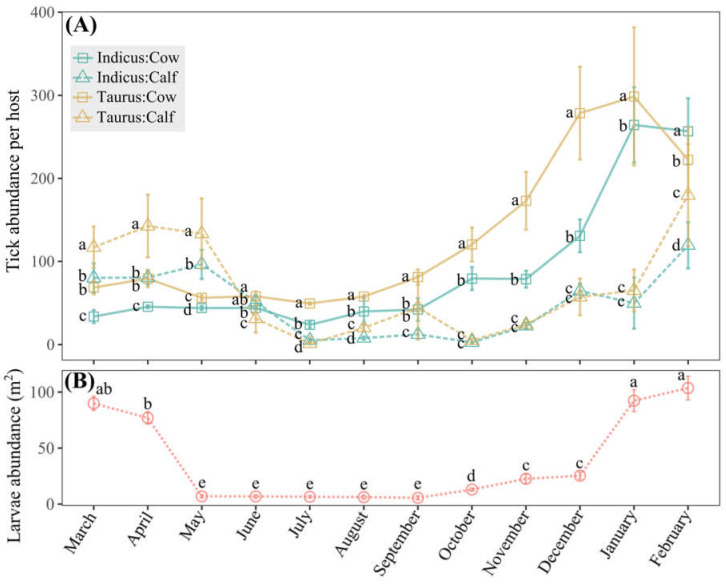
Dynamics of tick abundance in the Macagual study site. (**A**) Means of tick abundance of cows and calves of *B. indicus* and *B. taurus*, with monthly statistical comparisons of means. (**B**) Means of larvae abundance in grazing areas in a 12-month period, with comparisons made among different months. Means followed by the same letter do not differ according to the Tukey test (*p* < 0.05). Vertical error bars denote the standard error.

Monitoring tick abundance in cattle and larvae abundance in grazing areas over a year in the Macagual study site indicated population seasonal dynamics (Figure 5). *Taurus* cows experienced a significant rise in tick abundance starting in September, reaching a peak in January (Figure 5A and Supplemental Table S2). *Indicus* cows exhibited a less pronounced increase during the same period but still peaked in January. For calves of both breeds, the abundance remained relatively lower compared to cows throughout the year; however, their abundance trends were consistent with those observed in cows, suggesting a similar seasonal pattern (Figure 5A and Supplemental Table S2). The tick abundance on *B. taurus* cows was significantly higher (*p* < 0.05) than that on *B. indicus* cows during most months, with the highest recorded tick counts approaching 300 per individual (January) and the lowest approaching 50 per individual (July) (Figure 5A). Larvae abundance peaked dramatically in January, approaching 100 individuals per m^2^, and stayed low from May to September, varying between 6 and 7 individuals per m^2^ (Figure 5B). 

The observed seasonal variations in tick and larvae abundance led us to investigate their correlation with environmental factors that fluctuate throughout the year, as they may influence the life cycle and behavior of ticks. We performed a redundancy analysis (RDA) to elucidate the relationship between key environmental variables (temperature, rainfall, and relative humidity) and tick populations across cattle racial groups (*B. indicus* and *B. taurus*) in cows and calves, and larvae populations in grazing areas, over the annual cycle. 

**Figure 6:**
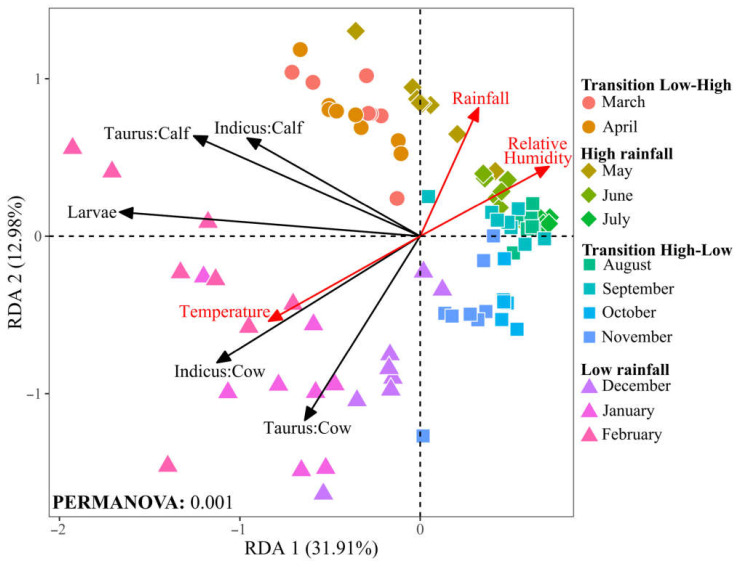
Redundancy analysis (RDA) of tick and larvae and environmental variables at the Macagual study site. Environmental vectors are plotted as red arrows. Black vectors represent the centroid of each group, reflecting the mean value for tick and larvae abundance. Data points indicate tick abundance in different cattle groups and larvae over the months, with shapes representing the seasonal rainfall patterns and colors representing months. Significance was determined through PERMANOVA analysis with 999 permutations.

Collectively, the first two canonical axes of the RDA accounted for 44.9% of the total variance, indicating a significative relationship between environmental factors and tick population dynamics (Figure 6). The RDA indicates a positive correlation between temperature and tick abundance, particularly in cows of both racial groups. This pattern suggests that the warmer conditions typical of the low rainfall season may increase tick populations. Conversely, rainfall and relative humidity negatively correlate with tick and larvae populations. This trend indicates that conditions of high humidity, characteristic of the high rainfall season, are less favorable for tick survival or proliferation. 

**Figure 7:**
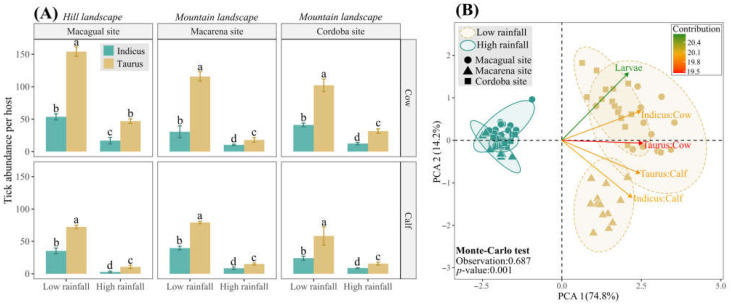
Tick abundance in hill and mountain landscapes according to rainfall seasonality. (**A**) Mean tick abundance for cows and calves of *B. indicus* and *B. taurus*, at the three sample sites: Macagual, situated in a hill landscape, and Macarena and Cordoba, located in mountain landscapes. Data are presented for both low and high rainfall seasons. Within each study site, tick abundance means for cows of both breed groups are compared, and separately, means for calves of both breeds are compared. Means followed by the same letter do not differ according to the Tukey test (*p* < 0.05). Vertical error bars represent the standard error. (**B**) Principal Component Analysis (PCA) of tick abundance and rainfall seasons projected on the ordination plane PCA 1/PCA 2 and the sample sites (shapes) grouped according to the rainfall season (colors). The color of the vectors indicated the contribution of the variable. Significance was determined through the Monte-Carlo test with 999 permutations.

Although most cattle livestock production is performed in the hill landscape of this region, it also extends to mountain areas. Therefore, we also assessed the tick abundance in cows and calves of both racial groups during low and high rainfall seasons and two farms, Macarena and Cordoba, located in the mountain landscape. Similar to our findings in the Macagual study site (hill landscape), we observed higher tick abundance in hosts of the *B. taurus* breed group, confirming their greater susceptibility to tick infestation (Figure 7A). Furthermore, we explored the relationship between tick abundance and rainfall seasons across hill and mountain landscapes using Principal Component Analysis (PCA). The first two components of the ACP explained 89% of the variance, with tick abundance grouped into two clusters aligned with rainfall seasonality (Figure 7B) (*p* < 0.001; 69% of explained variance). This result highlighted a consistent positive correlation between high tick abundances and low rainfall conditions across livestock production systems independently of the landscape in the Colombian Amazon region. 

## 4. Discussion

The prevalence of ticks in cattle livestock systems within equatorial zones is acknowledged due to the favorable environmental conditions conducive to their proliferation [4,7,40,41]. However, the current situation of tick infestation in the northwestern region of the Colombian Amazon, where the cattle industry is undergoing increasing economic significance, remains largely unexplored. Our investigation fills this gap by characterizing tick populations within livestock production systems in this geographical area.

Our findings indicate that *R. microplus* is the predominant tick species in the northwestern Colombian Amazon. This aligns with the well-documented association of *R. microplus* with cattle [6,7,8,18]. The species-specific host preference is critical in its prevalence within cattle populations across tropical and subtropical regions, including our study area. Although 46 tick species have been reported in Colombia [19], many may exhibit preferences for hosts other than cattle, which may explain their absence in our cattle-focused study.

The presence of unique haplotypes for the 16S rRNA and *COX1* genes across all examined specimens suggests limited genetic variation within the tick population of the northwestern Colombian Amazon, a phenomenon observed in other regions globally [8,42,43,44]. The observed lack of genetic differentiation may be attributed to factors such as commercial livestock migration patterns, which potentially restrict interactions between disparate tick populations [43,45], thereby diminishing opportunities for genetic exchange and promoting population homogeneity.

Phylogenetic analyses based on mitochondrial 16S and *COX1* gene regions, widely recognized as effective for resolving phylogenetic relationships within *R. microplus* complex and closely related species [7,8,43,46], indicated that the haplotype identified in our study, designated as *R. microplus* Caquetá Colombia, was positioned to *R. microplus* Clade A. This clade predominantly encompasses isolates from America, Asia, and Africa. Interestingly, our analysis indicated the same sequences from Colombia to the haplotype identified in our study for both the 16S and *COX1* genes. This proximity suggests a shared evolutionary history and genetic relatedness among tick populations from Colombia and those within the *R. microplus* Clade A. Future investigations will benefit from integrating multiple molecular markers or whole-genome sequencing analyses to advance our understanding of tick genetic structure and the evolutionary history of tick populations.

The observed seasonal variations in tick abundance, particularly in *taurus* and *indicus* cows, highlight the influence of seasonal dynamics on tick infestation severity. Notably, individuals of the *B. taurus* breed exhibited significantly higher levels (*p* < 0.05) of tick presence compared to their *indicus* counterparts. This finding aligns with previous studies, which have documented a higher susceptibility of *B. taurus* to parasitic infestation, attributed to genetic and physiologic differences between the breeds [47,48]. Factors such as a less effective immune response to tick antigens and differences in skin thickness and grooming behaviors may contribute to this disparity [49]. This susceptibility to parasitic infestations underscores the challenge posed by the poor adaptability of *B. taurus* to tropical environmental conditions characterized by high humidity and temperature [50,51,52]. To mitigate these issues, particularly within tropical regions and dairy production systems, adopting crossbred or synthetic breeds known for their heightened resistance to parasite infestations is recommended [53].

Furthermore, the difference in tick abundance between calves and adult cows suggests that host susceptibility and immune responses vary across different age groups. Previous research has shown that tick prevalence is influenced by factors such as inherited immunity status at birth (which is particularly relevant in Creole animals due to their innate immunity), the timing of separation from the mother, and differences in body surface area between young and adult animals [54,55].

The tick *R. microplus* is a single-host parasite, with larvae serving as the dispersal stage to reach hosts in the pasture. These larvae tend to congregate at the uppermost part of forage, where they are attracted by the carbon dioxide exhaled by grazing animals [56]. In our study, we exclusively identified *R. microplus* species in the pasture, confirming its status as the primary tick affecting cattle in the northwestern Colombian Amazon region. This finding aligns with previous research conducted not only in our region but also in Argentina, along the United States–Mexico border, and in various regions of Colombia, including Cundinamarca, Boyacá, and Bolivar [18,25,57,58]. Furthermore, our study highlights that the abundance dynamics of larvae in grazing areas positively correlates with the prevalence of ticks infesting the animals. This correlation highlights the critical importance of implementing comprehensive tick control measures, encompassing both parasitic and non-parasitic stages.

Our study elucidated a significant correlation (*p* < 0.001) between tick abundance and environmental factors such as temperature, rainfall, and relative humidity, all recognized as relevant for tick biology [12,13]. Positive associations between temperature and non-parasitic and parasitic tick phases, particularly among cows of both racial groups, suggest that warmer conditions may facilitate tick proliferation. Conversely, negative correlations with rainfall and relative humidity indicate that humidity levels typical of high rainfall seasons are less conducive to tick survival or proliferation. These results are consistent with previous field and laboratory studies linking environmental conditions to various aspects of tick biology, including egg incubation periods, larval pre-hatching, and larval longevity [59,60].

Future research could delve deeper into the mechanisms underlying host susceptibility to tick infestation and the impact of environmental factors on tick population dynamics. Additionally, exploring the efficacy of integrated tick control measures, such as strategic acaricide application and pasture management practices aligned with seasonal climate patterns, could provide valuable insights into sustainable tick management strategies in the Colombian Amazon region.

## 5. Conclusions

Our study identified a tick species within Clade A of the *R. microplus* complex as the predominant one affecting cattle in the northwestern Colombian Amazon. We observed limited genetic variation within the tick population, suggesting a uniform genetic makeup. Importantly, our findings reveal a significant positive correlation between tick abundance and warmer temperatures during the low rainfall season and a negative correlation during the high rainfall season. These seasonal dynamics suggest specific periods of vulnerability to tick infestations.

Based on these findings, we recommend targeted tick control efforts during warmer periods. This could involve strategically timing acaricide applications to coincide with peak temperature phases, which our data indicate are high-risk periods for tick proliferation. Additionally, adjusting cattle grazing times and improving pasture management during these warmer months could mitigate the risk of tick infestations. Implementing these integrated pest management strategies, considering both climatic factors and host characteristics, is essential for effective, sustainable tick control in the region.

## Data Availability

Data are available within this article and Supplementary Materials.

## Supplementary Materials

The following supporting information can be downloaded at: https://www.mdpi.com/article/10.3390/vetsci11060244/s1. Figure S1: Alignment of 16S rRNA gene sequences from *Rhipicephalus microplus* specimens belonging to Clade A; Figure S2: Alignment of COX1 gene sequences from *Rhipicephalus microplus* specimens belonging to Clade A; Table S1: Monthly environmental data at the Macagual study site; Table S2: Monthly Dynamics of Tick Abundance at the Macagual Study Site. Mean tick abundance values for each month, categorized by breed group (*B. indicus* and *B. taurus*) and age (calf and cow). Standard errors is presented in parentheses. Within each category, means accompanied by the same lowercase letter do not differ significantly, as determined by the Tukey test (*p* < 0.05).
