# Supplementary material for: Characterization and Seasonal Dynamics of Tick Populations in Dairy Cattle Production Systems of Northwestern Colombian Amazon
Source: Vet Sci. 2024 May 29;11(6):244. doi: 10.3390/vetsci11060244 (PMC11209389; doi:10.3390/vetsci11060244)
Supplement: Supplementary file 1 [file vetsci-11-00244-s001.zip › vetsci-2971689-supplementary.pdf]

veterinary sciences

MDPI

**Supplemental Table S1**. Monthly environmental data at the Macagual study site.

| Year | Month | Temperature (°C) | Rainfall (mm day$^{-1}$) | Relative Humidity (%) |
|---|---|---|---|---|
| 2020 | March | 29.0 | 204 | 84 |
| | April | 28.3 | 252 | 86 |
| | May | 27.3 | 249 | 88 |
| | June | 26.4 | 247 | 89 |
| | July | 26.3 | 224 | 88 |
| | August | 27.8 | 128 | 83 |
| | September | 29.0 | 116 | 81 |
| | October | 28.9 | 172 | 84 |
| | November | 28.5 | 210 | 88 |
| | December | 28.8 | 152 | 86 |
| 2021 | January | 29.7 | 94 | 79 |
| | February | 30.0 | 131 | 78 |

**Supplemental Table S2. Monthly Dynamics of Tick Abundance at the Macagual Study Site. Mean** tick abundance values for each month, categorized by breed group (*B. indicus* and *B. taurus*) and age (calf and cow). Standard errors is presented in parentheses. Within each category, means accompanied by the same lowercase letter do not differ significantly, as determined by the Tukey test ($p < 0.05$).

| Year | Month | *B. indicus* | | *B. taurus* | | Overall |
|---|---|---|---|---|---|---|
| | | Calf | Cow | Calf | Cow | |
| 2020 | March | 80.20 (35.86) bc | 33.80 (15.16) de | 117.00 (52.32) c | 68.80 (30.76) ef | 74.95 (16.75) e |
| | April | 80.60 (36.04) bc | 45.60 (20.33) d | 142.60 (63.77) b | 79.40 (35.50) e | 87.05 (19.46) d |
| | May | 96.40 (43.11) b | 44.00 (19.67) d | 133.40 (59.65) bc | 56.40 (25.22) fg | 82.55 (18.45) de |
| | June | 51.42 (19.43) de | 44.20 (19.77) d | 31.33 (18.09) fg | 58.20 (26.02) fg | 48.30 (10.80) fg |
| | July | 5.28 (1.99) hi | 23.75 (11.85) e | 1.33 (0.77) h | 49.50 (24.75) g | 18.55 (4.37) i |
| | August | 7.85 (2.97) gh | 40.00 (20.00) d | 20.33 (11.73) g | 57.83 (23.61) fg | 31.15 (6.96) h |
| | September | 12.33 (5.03) g | 42.20 (18.87) d | 44.50 (22.25) ef | 81.20 (36.31) e | 43.45 (9.71) g |
| | October | 2.71 (1.02) i | 79.40 (35.50) c | 5.00 (2.88) h | 120.40 (53.84) d | 51.65 (11.54) f |
| | November | 22.42 (8.47) f | 78.75 (39.37) c | 24.33 (14.04) g | 173.00 (70.62) c | 79.15 (17.69) de |
| | December | 64.71 (24.46) cd | 130.75 (65.37) b | 57.33 (33.10) de | 278.50 (113.69) a | 140.95 (31.51) c |
| 2021 | January | 49.83 (20.34) e | 264.40 (118.24) a | 65.20 (32.62) d | 298.60 (133.53) a | 168.75 (37.73) b |
| | February | 119.50 (48.78) a | 256.75 (128.37) a | 179.75 (89.87) a | 222.16 (90.69) b | 189.80 (42.44) a |

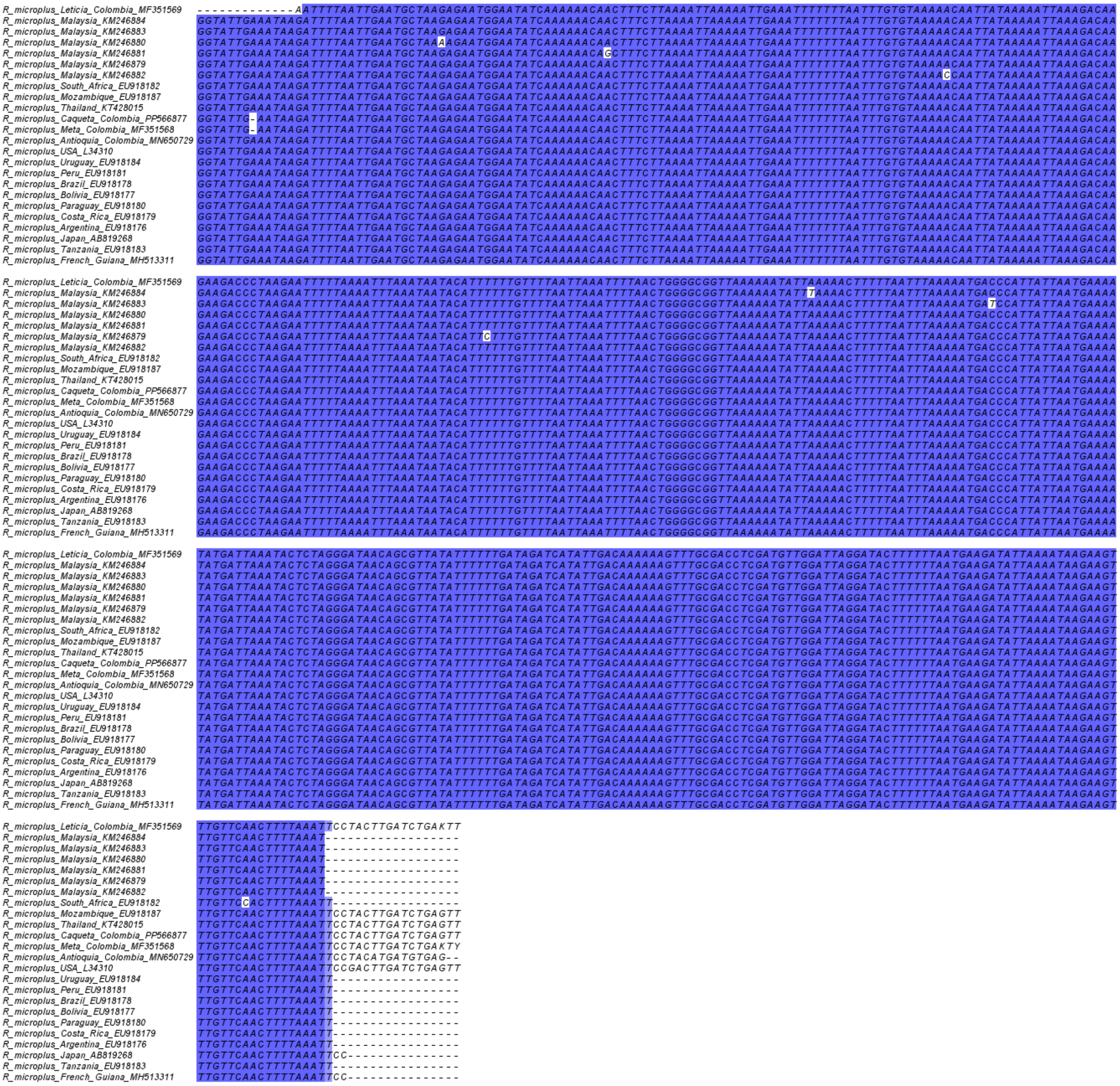

**Supplemental Figure S1.** Alignment of 16S rRNA gene sequences from *Rhipicephalus microplus* specimens belonging to Clade A.

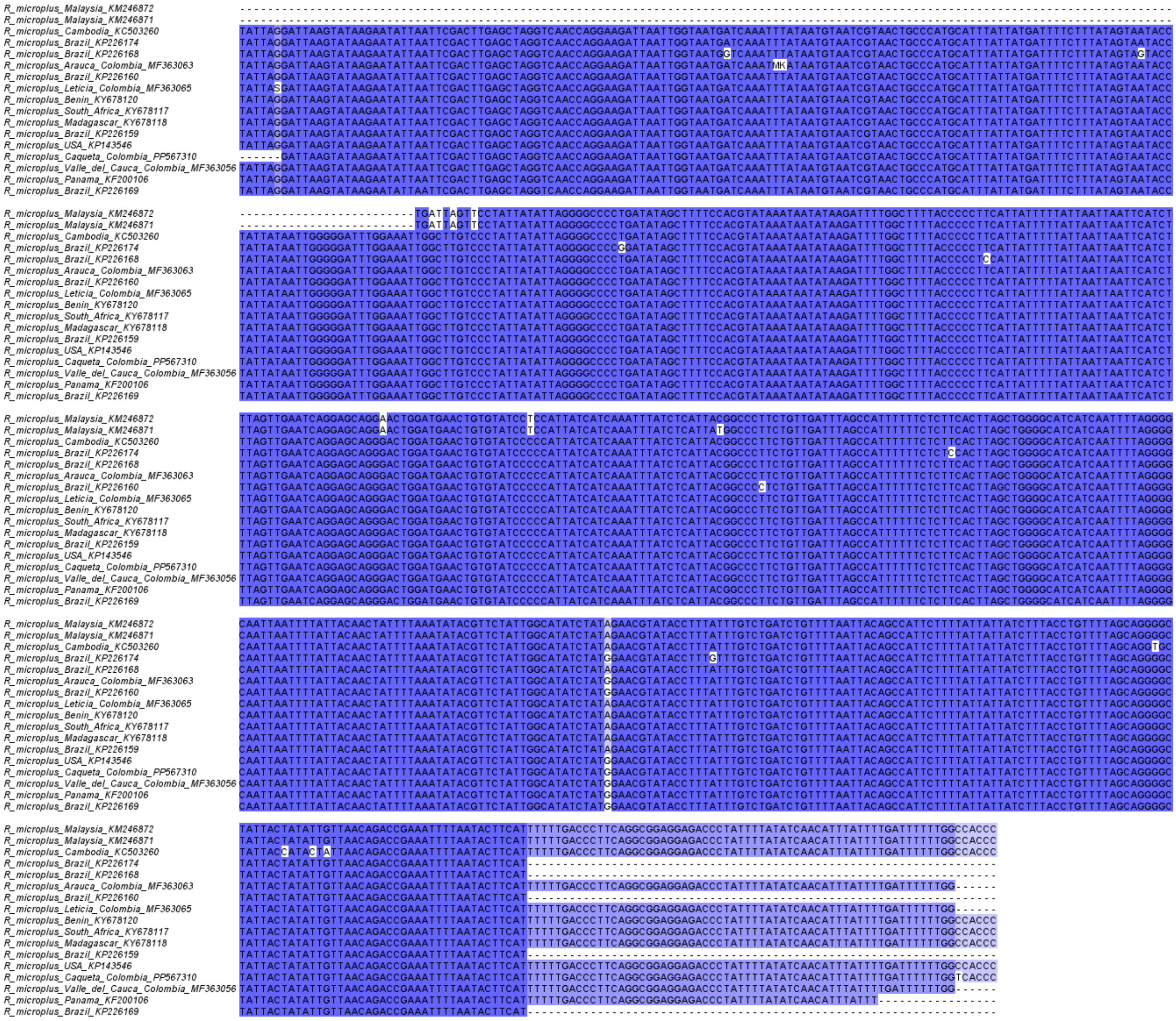


**Supplemental Figure S2.** Alignment of *COX1* gene sequences from *Rhipicephalus microplus* specimens belonging to Clade A.
